# Symbiota – A virtual platform for creating voucher-based biodiversity information communities

**DOI:** 10.3897/BDJ.2.e1114

**Published:** 2014-06-24

**Authors:** Corinna Gries, Edward E. Gilbert, Nico M. Franz

**Affiliations:** † University of Wisconsin, Madison, Madison, United States of America; ‡ Arizona State University, Tempe, United States of America

**Keywords:** Biodiversity informatics, digitization, economy of scale, natural history collection, Open Source, virtual collection portals

## Abstract

We review the Symbiota software platform for creating voucher-based biodiversity information portals and communities. Symbiota was originally conceived to promote small- to medium-sized, regionally and/or taxonomically themed collaborations of natural history collections. Over the past eight years the taxonomically diverse portals have grown into an important resource in North America and beyond for mobilizing, integrating, and using specimen- and observation-based occurrence records and derivative biodiversity information products. Designed to mirror the conceptual structure of traditional floras and faunas, Symbiota is exclusively web-based and employs a novel data model, information linking, and algorithms to provide highly dynamic customization. The themed portals enable meaningful access to biodiversity data for anyone from specialist to high school student. Symbiota emulates functionality of modern Content Management Systems, providing highly sophisticated yet intuitive user interfaces for data entry, batch processes, and editing. Each kind of content provision may be selectively accessed by authenticated information providers. Occupying a fairly specific niche in the biodiversity informatics arena, Symbiota provides extensive data exchange facilities and collaborates with other development projects to incorporate and not duplicate functionality as appropriate.

## Introduction

For more than 250 years biodiversity has been documented in the form of specimens deposited, curated and maintained in over 1600 natural history collections in the United States. More than 1 billion U.S. specimens are widely used in scientific research, land management decision making and education (Suarez and Tsutsui 2004). Information associated with those specimens and collated into large data products can allow wider use of this massive body of knowledge to answer new research questions, provide better decision support, and broaden access (Graham et al. 2004; Lister, Adrian M. and Climate Change Research Group 2011). Tremendous value is added by enabling analytical methods developed in the "Big Data" realm (Marx 2013). However, digitizing this information is a massive undertaking that needs large-scale financial and technical support.

Several approaches have been developed taking advantage of technologies for reducing repetitive work of digitizing collections and improving the quality of data. Each of the technologies currently in use and providing varying degrees of functionality for managing collections, digitizing specimens, providing on-line access to the records, or supporting large-scale analyses has been developed by and for different user communities and with slightly different emphases on the above mentioned functionalities; e.g., Arctos (http://arctosdb.org/), KE Emu (http://emu.kesoftware.com/), SilverBiology (http://www.silverbiology.com/), Specify (http://specifysoftware.org/), or VertNet (http://vertnet.org/).

This paper provides an overview of one such framework, the Symbiota software platform for networking biodiversity data (Gilbert et al. 2014; http://symbiota.org), with emphasis on the software's design principles and functionality. With an estimated total of more than 350 participating collections, 2500 active data providers, and 12+ million digitized specimen records spanning major organismal groups (fungi, lichens, plants, invertebrates, vertebrates), Symbiota is a leading Open Source platform in North America for mobilizing, integrating, and using specimen- and observation-based occurrence records and derivative products. Symbiota has prospered in recent years, as mirrored by a rapidly diversifying user base and its important role in supporting digitization efforts within the United States National Science Foundation's Advancing Digitization of Biological Collections program (NSF ADBC 2011b). The latter was launched in 2010, with annually recurring competitions, to create Thematic Collections Networks (TCNs), i.e., "collaborations among collections that are organized and justified by a scientific question" (NSF ADBC 2011a). Of the ten TCN projects awarded in the past three years, five critically depend on Symbiota to achieve their objectives (iDigBio 2013). Four additional Partner to Existing Network (PEN) proposals (out of a total of seven) were awarded to enhance these sponsored Symbiota networks. Hence the apparent match between Symbiota's design and nationwide aims to advance the digitization of natural history collections through mission- and project-centered initiatives (NIBA 2010; Baker 2011; Hanken 2013) is noteworthy.

In what follows, we concentrate on Symbiota's overarching principles, functions, and current and future applications. More detailed information on the functionality of particular modules (such as the interactive keys) will be treated elsewhere. We begin with reviewing the history of Symbiota's origins which has shaped the overall design with regards to conceptual, technical, and sociological aspects. We then discuss modules for managing specimen and/or observation occurrences, biotic inventories, and identification keys each from the perspective of general portal users and contributors. We close with an overview of Symbiota's current use and acceptance. Throughout, we attempt to explain *why* Symbiota has been embraced by its user communities, and lay out design and functionality trade-offs with relevance to larger-scale trends in biodiversity informatics (Thessen and Patterson 2011; Wheeler et al. 2012; Hardisty et al. 2013).

### Symbiota design principles – history and conceptual aspects

The history of pre-Symbiota development dates back to early digitization efforts and local solutions for database supported collections management. Early technical collaborations involved the HyperSQL project (Newsome et al. 1997), the Specimen Management System for California Herbaria (SMASCH; Moe 2000), the Central Arizona Phoenix Long-Term Ecological Research Site  (CAP-LTER; http://caplter.asu.edu/; Grimm and Redman 2004) and its information management system, and particularly the Southwest Environmental Information Network (SEINet; Schoeninger et al. 2002; McCartney 2003; McCartney et al. 2003). The *user community* – e.g., collections managers, taxonomists, ecologists, data entry personnel, programmers, informaticians, and students – have driven much of the overall software design philosophy and implementation.

An impactful award made in 2008 led to an increasing separation from the initial general data management of Symbiota, initially as an independently designed MySQL/PHP identification tool working off of user-generated checklists. Subsequent redesign and enhancements led to the integration of a Darwin Core-based (Wieczorek et al. 2012), centralized collections management module which allowed the creation of multiple portals for diverse taxonomic domains.

In its current realization, Symbiota is designed to support and promote grassroots bio-collaborations that work towards efficient data mobilization, improving data quality, and describing biodiversity in the form of virtual floras and faunas. Of particular importance is the emphasis on mobilizing information in order to address specific research questions, where the primary data are distributed among numerous collections. Collections are regarded as scientifically interdependent. The need to address shared *research* objectives takes primacy in software development over the more traditional design focus on customizable collection *administration* (e.g., handling in-house locations and curation trajectories of physical resources or processing loans). Although the focus on strengthening small grassroots collections collaborations still characterizes Symbiota's primary niche in the biodiversity data environment today, its modular design has enabled Symbiota to nimbly respond to changing needs and requirements, now supporting entire specimen digitization and collections management workflows plus extensive data exchanges with other systems.

**Collaboration eases individual efforts and leads to enhanced information quality.** Symbiota's primary focus is on permanently vouchered occurrence records of high scientific quality and accountability and on providing the tools to *collaboratively* manage this information. The platform has a modular framework for publishing biodiversity information; i.e., natural history collection occurrence records or observations, taxonomic information, images, species profiles, and taxon character and character state information in interactive keys for identification. Access to modules is achieved by emulating the functionality of a modern content management system (CMS). The system promotes a *positive feedback loop* that includes: (1) making data public instantaneously which can serve to expose errors; (2) using web messaging to alert responsible parties to such errors; (3) using web-based editing tools and workflows that allow such errors to be resolved as they are identified; (4) redirecting data repairs back to a source collection's internally used platform; and (5) rendering repairs permanent at the broader scale with the subsequent data update. In short, Symbiota leverages a themed, collaborative biodiversity data mobilization approach towards improving the quality of individual collections' data (Costello et al. 2013).

The CMS approach enables assignment of specific user permissions to share the tasks of data build-up and management of central resources. For instance, designated taxonomy coordinators may be responsible for the taxonomic thesaurus (see details below) to retain community-level acceptance and assure regular updates fostering the collaboration between taxonomic and regional experts. Symbiota also facilitates extensive data exchange options with other systems. For instance, collections record data may routinely be provided to the Global Biodiversity Information Facility (GBIF), taxon descriptions can be exchanged with the Encyclopedia of Life (EOL), and data may be synchronized with local management systems.

**Portals encourage participation through shared branding and diversified forms of engagement.** Symbiota can leverage and enrich biodiversity data through the creation of *portals* led by thematically coherent *research communities.* These communities tend to have shared taxonomic and/or geographic concentrations; and consequently shared interests to work collaboratively towards virtual floras and faunas that constitute authoritative, high quality vouchered treatments of a region's biota. In tailoring towards such communities, Symbiota has become a biodiversity information platform which is configurable, customizable, and independently manageable by each research community, and thus analogous to the Scratchpads approach (Smith et al. 2011).

**Figure 1.**
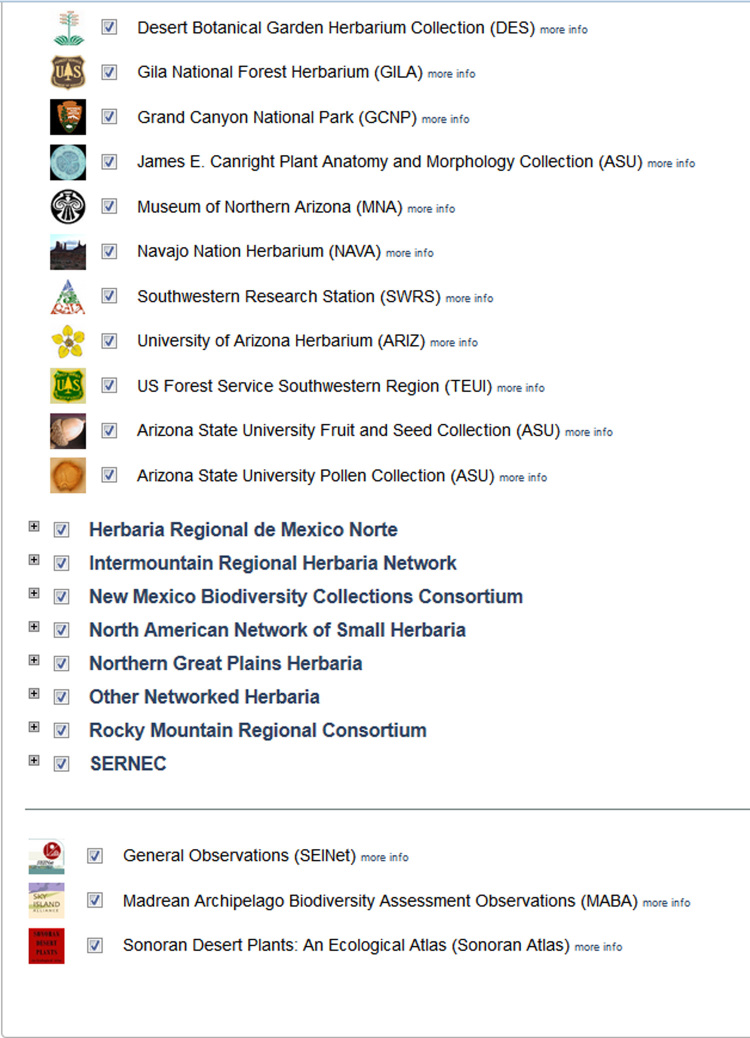
Partial screenshot of the Search Collections panel for SEINet, showing Symbiota's ability to integrate the identities of individual member collections (with their respective logos and links to portal-configured homepages), regional portals (only four out of nine portals shown here), and multi-portal 'hubs'. All screenshots used in this paper were taken in February 2014.

**Figure 2.**
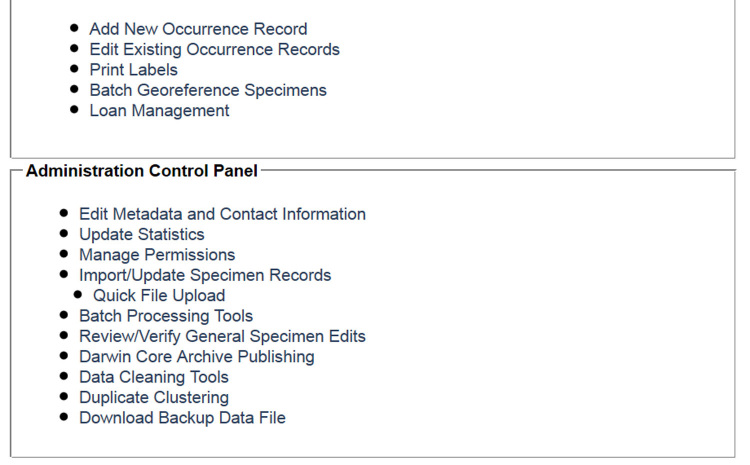
Screenshot of the Collection Management Panel for administrators of the University of Wisconsin Lichen Collection. The Data Editor Control Panel facilitates most day-to-day tasks related to specimen digitization as well as loan activity, whereas the Administration Control Panel focuses on managing the collection's appearance in the portal.

Each web-based portal allows a wide range of forms of engagement for consortia, institutions, collections, research teams, individual researchers, and public groups or citizens (Fig. 1). Collections have the option to participate in Symbiota portals through either direct "Live Data" management or a "Data Snapshot" harvested periodically from another database system. In the former case, Symbiota's Collection Management system is used directly to achieve day-to-day digitization and biotic inventory tasks (Fig. 2); whereas in the latter case the portal is set up to merely harvest and expose information that is generated and edited in another, locally preferred software environment. Symbiota has a diverse suite of data interoperability features to allow for such flexibility; including manual or API-supported, Darwin Core-compliant (Wieczorek et al. 2012) data ingestion and extraction, interactive data field mapping, and standard and custom cleaning of mapped uploads. The enhanced data upload/download features and option to select live versus snapshot engagement jointly lower the threshold for engaging new member collections that may have existing commitments to other platforms. Over time, a lowered threshold for engagement accelerates portal growth.

Symbiota furthermore allows individual collections to partake in multiple portals with overlapping thematic orientations. For instance, Arizona State University's Vascular Plant Herbarium is a member of both SEINet (Live Data) and Cooperative Taxonomic Resource for American Myrtaceae portal (CoTRAM; Data Snapshot), contributing only a subset of its full dataset to the latter. The flexibility offered in shaping and redirecting a collection's multiple virtual portal identities and thereby prioritizing one or more distinct biodiversity themes can enhance multi-portal growth and connectivity with minimal added effort.

**Figure 3.**
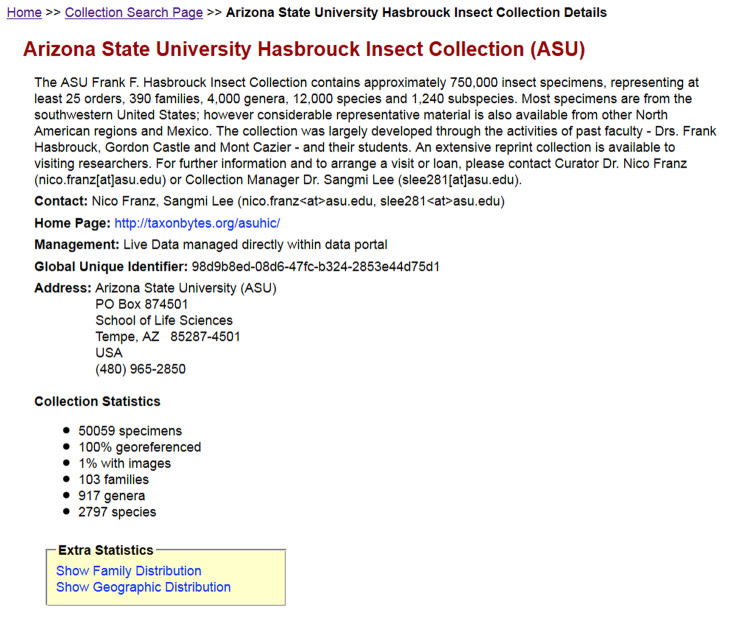
SCAN portal homepage for the Arizona State University Hasbrouck Insect Collection, which is being managed "Live" in SCAN, with an account of the collection's holdings, contact information, and Collection Statistics as of December, 2013.

Although the collaborative approach is emphasized, portals maintain the *provenance* of information and content authorship. All occurrence records and images in Symbiota are indelibly tagged to their source collection, via a collection code and/or specific icon. Each collection, in turn, maintains a separate portal identity and homepage that provides a summary of its holdings, members, additional contact information, and collection statistics – number of specimens, percentage of georeferenced specimens, images, number of species, etc. (Fig. 3).

**Figure 4.**
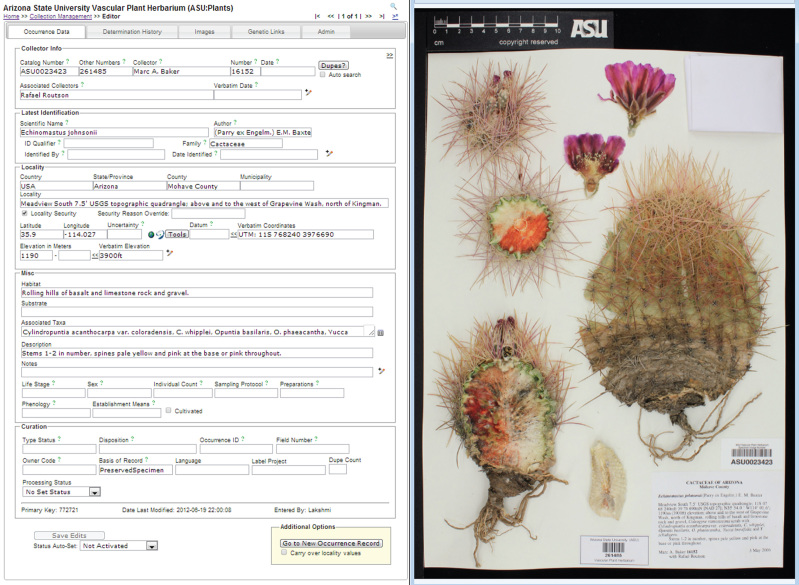
Screenshot of the occurrence data form corresponding to the SEINet specimen ASU0023423 – *Echinomastus johnsonii* (Parry ex. Engelm.) E.M. Baxter (common name: Johnson's fishhook cactus – pertaining to the ASU Vascular Plant Herbarium collection. This is the primary Symbiota contributor interface through which individual specimen records are entered and edited. At the highest level, contributors can switch from the Occurrence Data tab to the Determination History, Images (see also right half), Genetic Links (e.g. to GenBank; Benson et al. 2012), and Administrative tabs. Entry personnel can zoom in on the label. Many data fields have inherent auto-completion or uniqueness/compliance checking functions, or can be expanded via the +-pencil icon for more fine-scale data entry. The Latest Identification data field section is linked to the taxonomic thesaurus, thereby ensuring correct integration of the Scientific Name with the portal-level taxonomy. Georeferencing support tools including Google Earth mapping and an embedded GEOLocate module. Under Curation, the Processing Status may be set to (e.g.) "Pending Review" to support filtering and data quality control practices among collection members.

**Modularization facilitates customization while maintaining data integrity.** Symbiota strikes a balance between allowing portal communities to acquire distinct identities and functions while ensuring database integrity and consistency. The aim to network and integrate biodiversity data meaningfully sets limits to the degree to which portal configurations can vary. These limitations are most apparent in the format of the single *occurrence data* table (Fig. 4) which varies minimally across portals and only in data fields not mandated by the Darwin Core. The occurrence data table is furthermore linked to a portal's *taxonomic thesaurus*; i.e. a continuously updated, (yet also) unifying reference classification with valid and synonymous names that subtends all taxonomically based search functions and output displays. Contributors are not required to conform to conventions that are in flux (e.g., taxonomy), however the system encourages movement toward more unified information alignment. While joining a Symbiota portal requires a pragmatic acceptance of these relatively stable and constrained data formatting and taxonomic content conventions, the prospect of wide-ranging collaboration also creates a motivation to depart from an individual collection's 'optimal' solution regarding (e.g.) data formats, content, and display options. In short, Symbiota is not designed to facilitate 'limitless customization' of underlying database tables or functions, instead mandating a level of homogeneity that yields consistent information output across collections and portals and promotes best practices.

Beyond sharing core biodiversity data formats and tables, Symbiota portals *are* customizable in numerous ways that suit specific performance and engagement needs of the corresponding communities. The concept of modularity applies to both the process of developing the software and the actual application instances. This approach promotes interoperability and extensibility. Modularity is manifested at different levels, as follows: (1) Application modularity – the modules for managing specimens, biotic inventories, identification keys, and species profile pages are designed to function independently of one another. Additional modules such as those supporting label image transcription (e.g., crowd sourced transcription, Optical Character Recognition, and Natural Language Parsing) or remote specimen identifications are turned on or off according to a portal's needs. Custom front-end frames, logos, texts, rotating images, and interactive identification games represent additional configuration options. (2) Data modularity – a portal's member collections are represented as independent units, each with its own management regime (Fig. 2). At the same time they are fully integrated through the underlying Symbiota database installation. (3) Portal modularity – several portals such as SEINet, the Intermountain Regional Herbarium Network, SERNEC, and the Madrean Archipelago Biodiversity Assessment are branded as separate portals by their geographically defined project themes, yet they all share a single data source supplied as a network of replicated databases.

Modularization also means deploying existing web services and workflows that are optimized for specific functions instead of creating them anew. Symbiota subscribes to the 'small pieces loosely joined' tenet (Weinberger 2003). This means, for instance, that services related to mapping occurrence records are provided through Google Maps (https://maps.google.com/) whereas georeferencing tasks are supported by GEOLocate tools (Rios and Bart 2010). Voucher images can be imported – with proper accreditation – from numerous external sites such as the Encyclopedia of Life (Wilson 2003), Morphbank, or distributed storage managed by the owning institution. An Optical Character Recognition module for scanned herbarium labels utilizes the Tesseract-OCR engine (http://code.google.com/p/tesseract-ocr/) in combination with SALIX for label text parsing (Barber et al. 2013; Lafferty 2012; Lafferty and Landrum 2013). The Lichens, Bryophytes and Climate Change TCN (LBCC) creates a label parser specifically tuned for lichen and bryophyte labels. A separate module designed to facilitate specimen related communication employs Filtered Push annotation technology (Morris et al. 2013). Such internal or external services are called upon flexibly by Symbiota to ensure a wide range of functionalities while at the same time limiting the platforms size and complexity. The modules interconnect, but their development is distributed.

### Technical design aspects

Symbiota adheres to the Open Source paradigm (http://opensource.org/osd.html; see also Molloy 2011). All software programs required to set up and operate new Symbiota portals are openly and freely available. Portal installation and data population instructions are provided via the Symbiota software project website (http://symbiota.org). The Symbiota code base is regularly updated and available through a subversion repository (SVN) hosted by Source Forge (http://sourceforge.net/projects/symbiota/). Although Sourceforge has been the repository of choice, as the user and contributor community grows the code may be moved to GitHub (https://github.com) for easier access and communication among developers. The Symbiota CMS is written uniformly in the server-side PHP integrated with client-side JavaScript, with a MySQL back-end database. Any PHP compliant web server can be used, yet the software has been most thoroughly tested using Apache HTTP Server.

Symbiota is *exclusively* web-based, which means that all portal data as well as management and user functions are accessible from any stationary or mobile device with a modern web browser and internet access. The software design philosophy parallels that of a Content Management System (e.g., Drupal or MediaWiki) specifically for the biodiversity community. If development of Symbiota began today a system like Drupal could be used as a starting point to implement Symbiota's functionality, however, this was not available when development on Symbiota started. Symbiota's central data model and algorithms implement principles of inheritance, hierarchy, and encapsulation, as found in object-oriented programming and ontology research (Cheney et al. 2009; Morris et al. 2013). This approach reduces efforts of data entry and maintenance while making information access highly dynamic and improving user satisfaction.

Symbiota uses a centralized, server-based infrastructure solution for multiple parallel or inter-dependent information communities. The centralized model enhances performance and furthermore answers directly to the needs of many natural history collections; in particular medium- or small-sized collections with limited access to IT personnel and infrastructure (Scoble 2010; Hanken 2013; Hardisty et al. 2013). The challenge of constant upkeep and integration of physical server infrastructure, software and databases, data formats, and content is restricted to a single portal managing entity in the network; e.g. a major research university, museum, or long-term funded biodiversity informatics resource such as iDigBio (Matsunaga et al. 2013). The portal-managing node assumes the responsibility for technical support of the network. All other portal member collections and all contributing individuals are freed from these tasks.

### Using Symbiota portals

In this section we describe functionality that any user without login credentials may access in a Symbiota portal (see also Landrum et al. 2010; Franz et al. 2014b). A simple name/e-mail account creation and login process that requires no human approval will grant users additional permissions to comment on records, maintain their own private or public species lists, and contribute to data transcription efforts.

**Figure 5.**
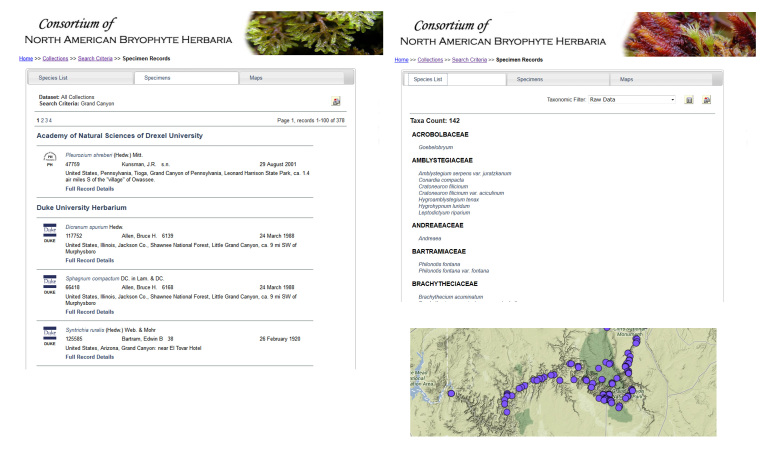
Screenshot of results displayed based on the search criteria ‘Grand Canyon’ which returns 378 records in 142 taxa. The Specimens panel (center tab) shown on the left presents an abbreviated summary view for each record that is expandable (not shown here). A Species List (left tab), shown on the right and Maps view (right tab, unselected), shown on bottom, are also available. A data icon in the top right corner facilitates downloading of the entire search results in Darwin Core or Symbiota CSV text file format (no permissions required). High-density occurrences of records are 'integrated' at coarser geographic scales and become resolved into separate latitude/longitude points at finer levels.

**Dynamic flora and fauna checklists.** Symbiota is designed to replace traditional, static floras or faunas with a *dynamic* approach. Modules are provided for each information concept found in such tomes – checklists, keys, taxonomic treatments and taxon descriptions with distribution maps and images. Linkages between the information content of each module provides for dynamic and user-driven data retrieval. Natural history specimen and (where appropriate) observation records are the core of any Symbiota portal, stored centrally in a relational schema based on the Darwin Core standard (http://www.tdwg.org/standards/450/). A standard specimen search engine with auto-completion and pick-list functionality facilitates taxonomic and geographic searches. Returned is a list of specimen records that match the criteria (Fig. 5) and which is instantly downloadable. The search also returns a taxon list for the chosen parameters; for instance, "Flora of Arizona", "Bats of the Phoenix Metropolitan Area", or "Lichens of the Grand Canyon". Any such checklist may be used within Symbiota to dynamically generate other components of a flora or fauna; i.e., an interactive key, taxon descriptions, distributions and images. The rapidly deployable on-line feature based on actual specimen information is largely unique to this platform and can support answering many research questions, as well as determining under- or un-collected regions.

**Figure 6.**
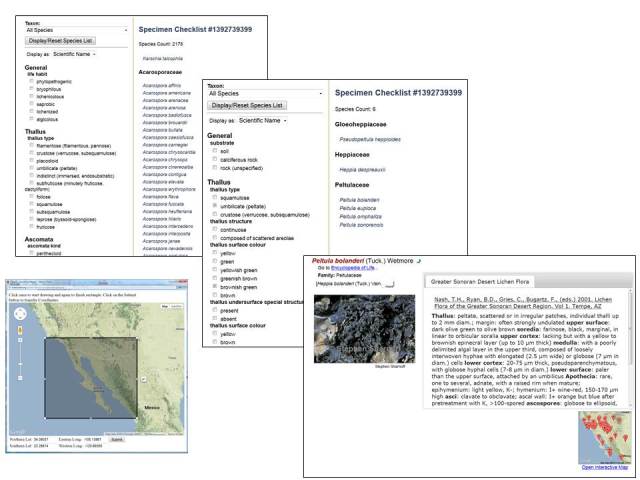
Screenshots of several stages in an exemplary sequence of using the Dynamic Key function in Symbiota. (A) Using the Dynamic Map interface, a rectangle is selected; the coordinates correspond to the bounding box are selected. Users can restrict the subsequent checklist creation process ("Build Checklist") through specification of a Taxon Filter (i.e., higher taxa down to family, listed alphabetically). (B) Using these search criteria, the module will search vouchers in Symbiota that may satisfy these conditions, and integrate the pertinent voucher list into a taxon (family/species) list with 2178 species-level matches. The Dynamic Key interface (left menu) is initially simple, including choices regarding e.g. habit. (C) In this example, selecting only two criteria from the list will (1) reduce the count of taxon matches to 6 and (2) display a remaining taxa-specific list of traits suitable for further determination, drawn from the character inheritance hierarchy of the corresponding key in Symbiota. These can either be further scrutinized with the dynamic key or determined through viewing of individual species profile pages.

The main feature of the **interactive keys** is the capability to dynamically assemble character and character state information for any given taxon group combination. The module can handle vastly different sets of characters for disjointed taxonomic groups within a single dataset. It furthermore optimizes displaying only those characters and states that differentiate taxa under consideration at any given step in the identification process. With every decision a user makes during the keying process, characters and states are added and/or removed, starting with more generally applicable characters and gradually moving towards specialized traits (Fig. 6).

Symbiota provides attractive **taxon profile pages** with natural language descriptions as well as images for each taxon. An occurrence map generator utilizes information from the collections records to dynamically produce Google distribution maps. Links to external resources (such as the Encyclopedia of Life) and Google searches for additional web resources are supported.

**Figure 7.**
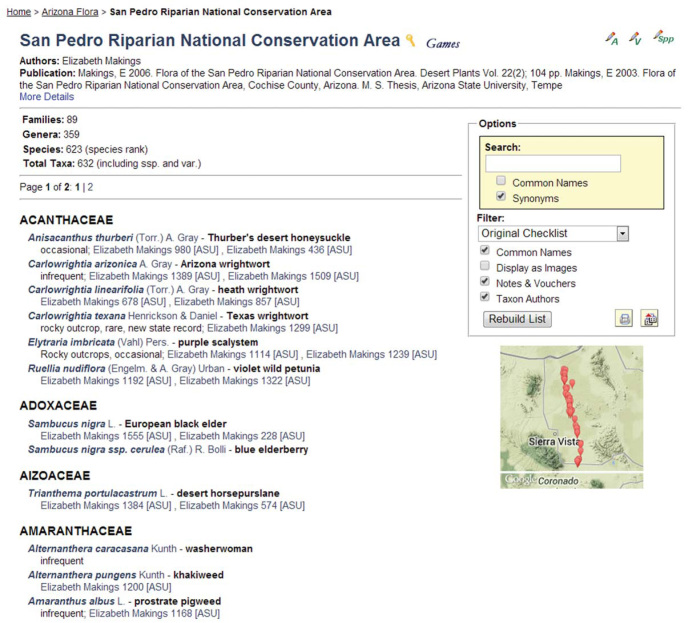
Screenshot of the homepage of the SEINet-derived San Pedro Riparian National Conservation Area checklist, a 'member' checklist of the Arizona Flora biotic inventory 'family' (Makings 2006, 2014). Checklist administrator functions are available in the top right corner ("A" – Administration, "V" – Manage Linked Vouchers, "Spp" – Edit Species List). The selected screenshot shows the entire list in alphabetical order, Taxon Authors, Common Names, and Notes & Vouchers. Clicking on vouchers listed for each taxon will display specimen details including images, when available. A Google Map thumbnail can be clicked for a more expansive map view.

The concept of virtual floras and faunas is best realized through Symbiota's **biotic inventories.** Such inventories are usually developed by an expert or an expert team, frequently starting with or leading up to a published taxon checklist. While these lists (and, hence, floras and faunas) may be generated dynamically from occurrence records (Fig. 6), well managed *static lists* have greater potential for accuracy and completeness (Fig. 7). Symbiota offers a flexible interface for exploring pre-compiled information for a target region (see also http://swbiodiversity.org/seinet/checklists/checklist.php?cl=3&showvouchers=1). Users can filter these lists by family, genus, species, or even common name. Results can be displayed with species authors, common names, habitat information, and/or voucher details. A list can be displayed as set of thumbnail images, thus making it easier to visualize the flora and/or fauna represented in the region. Static species lists can be integrated with multi-state identification keys, flashcard quizzes, or other tools and games.

In contrast to traditional print publications, these on-line checklists are fully rooted in voucher data records. A suite of management tools are available to build checklists, link vouchers, and coordinate species lists with herbarium level specimen annotations that result in the correction of misidentified vouchers. Maintenance of a set of checklist is facilitated by the ability to organize lists in a hierarchical relationship where parent checklists automatically inherit taxa and vouchers from all children checklists. This is particularly useful for creating integrated state and county lists; e.g., as currently in use by Park Service employees in the southwestern United States. Mangers can keep track of area-specific vouchers without the need of explicit collection level permissions from each herbarium holding the specimens. The hierarchical relationship of the checklists results in consolidated lists and reports available to the regional managers that are based on the independent efforts of a network of local data managers.

### Contributing to and maintaining Symbiota portals

Below we offer an overview of Symbiota features available to data contributors who have been granted data editing rights beyond those of general users. The platform supports wide-ranging interconnections among management tasks, resources, modules, and the resulting floras and faunas produced by Symbiota portal contributors. Additional information regarding Symbiota's data management functions is available at http://symbiota.org/docs/symbiota-data-management-tools/. Each Symbiota module provides consistent editing interfaces employing intuitive icons and tabs.

**Portal content management. User permissions** allow for task-specific access control. For each portal, highest-level access rights are typically confined to a small group of portal-level *super administrators* who can assign initial rights to collection and project leaders. From that highest level, additional rights assignments can cascade down to the individual collection and project domains. This promotes arrangements where no single person has overwhelming responsibilities to manage rights, and no single-point bottlenecks are created in developing a portal's contributor community. 'Global' or 'local' portal leaders can manage rights of their collection and project members at the appropriate levels.

In the **taxonomy managementmodule,** portal contributors with appropriate *taxonomy editor* rights can edit names, add names, undertake taxonomic rearrangements, change existing or provide new synonymy information, eliminate names that are in error, and modify the locality security settings for sensitive taxa. Interactive and flexible batch upload functions are available which permit rapid population of the taxonomic thesaurus with large classifications rendered in compliant file formats. Typically, such mass taxonomy population tasks are assigned to the *portal manager*. The module also allows the development and selection of alternative mappings among valid names and synonyms, reflecting (e.g.) parallel classification schemes such as the Flora of North America (http://www.efloras.org/flora_page.aspx?flora_id=1) and the USDA Plants Database (http://plants.usda.gov/).

Symbiota's **collection management module** is appropriate for managing collections entirely on-line and inside the Symbiota portal framework. The module has two control panels to enable *data editing* and *administration* (Fig. 2), respectively. In the former, searching for occurrence data records is facilitated through a multi-field *record search form* that serves up data through either an individual (editing) or tabular (browsing) display. The main occurrence data entry tool (Fig. 4) provides numerous user-friendly functions including auto-completion, instantaneous consistency/error checking for standardized names and formats, internal and external georeferencing support (e.g., via Google Maps and GEOLocate), and annotations related to the quality and curation status of the record. The system keeps track of all general specimen edits, thus allowing collection managers to view a complete history of changes made to any record. Additional tabs handle the record's determination history, other annotations, images, links to related genetic data, and other administration functions. The data editor control panel (Fig. 2) is rounded out with functions for label printing and innovative batch georeferencing and loan management modules that make use of information from and participation by other portal member collections, thus leading to a complete provenance history of a record's location and state of identification within the virtual portal community.

The data editor module supports workflows for digitizing label information from a specimen image. The label image (or label part of the image) is made available to the editor where Optical Character Recognition (OCR) and automatic parsing of this information into database fields (Natural Language Processing, NPL) may be applied. Voice recognition is currently in the experimental stage and may become available in the future for entering data. If a duplicate record was previously entered by a different institution, the system has the ability to quickly retrieve and import this information into the data entry form, thereby avoiding the need of retyping the same data for each duplicate specimen. This is a powerful feature for saving time and reducing errors in new entries as well as the existing one as they are collaboratively edited.

The administration control panel (Fig. 2) allows collection leaders to manage their collection's profile, statistics, permissions, mass specimen imports or updates, other batch processing, a complete and configurable provenance log of *all* occurrence record edits, Darwin Core Archive (DwCA) publishing (Remsen et al. 2011), data cleaning, duplicate clustering (e.g. for multiple herbarium specimens derived from an individual plant), and data back-up functions.

**Biodiversity data products.** Symbiota's interactive, multi-entry **identification keys** are generated directly from descriptive data stored in a relational data representation of the DELTA data standard (http://delta-intkey.com/). Such keys have many advantages over traditional, dichotomous keys (Dallwitz 2000; Brach and Song 2005; Brach and Song 2006; Hagedorn 2005; Hagedorn 2007; Thiele 2005; Brach and Boufford 2011). Nearly four decades of development of computerized, multi-character identification tools (Dallwitz 1974; Dallwitz 1980; Dallwitz 1992; http://www.lucidcentral.com/en-us/home.aspx; Dmitriev 2013) have advanced these tools to the point where assessing only 2-5 characters and states can often reduce a long list of candidate species to a manageable size (Fig. 6). The low-level identification of taxa can be attainable in a short time by subsequent browsing of images, distribution maps, and descriptions (Edwards and Morse 1995; Hagedorn et al. 2004; Hagedorn et al. 2005).

Symbiota's novel integration of taxonomic *and* distributional information (Fig. 6) greatly accelerates the keying process while also rendering the character/state entry and maintenance tasks more efficient. The management interface is based on the hierarchy defined within the taxonomic thesaurus, thus facilitating the implementation of character inheritance. Since genera inherit family-level characters and species inherit genus-level characters, only the features that distinguish a species from its close relatives need to be entered. Character reversals and polymorphisms are allowable in the system. Keys do not need to be broken down into several levels of complexity; users can proceed from one key (rank) level to the next even if a large region or taxonomic group is treated. Characters may be added or deleted at any time without compromising the integrity of the key. Moreover, the module can accommodate cases where different taxonomic groups utilize the same terms to denote different morphological structures. A more detailed account of Symbiota's interactive key feature will be provided elsewhere (Gilbert et al., in preparation).

**Figure 8.**
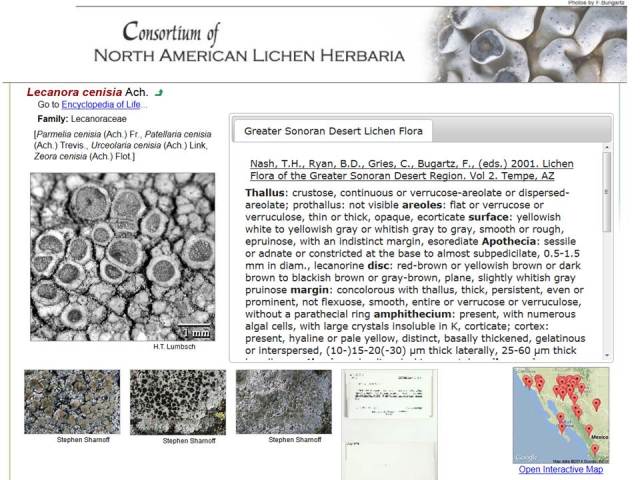
Screenshot of the Species Profile page of *Lecanora cenisia*. Ach. (Lecanoraceae) in CNALH (http://lichenportal.org/portal/taxa/index.php?taxauthid=1&taxon=53780&cl=2), with an outlink to the corresponding Encyclopedia of Life page (http://eol.org/pages/196725), common names, an assortment of accredited and clickable thumbnail images including *in situ* photographs and herbarium voucher scans (additional images and links are displayed when clicking links below), an Interactive Map for documented species occurrences, and taxonomic diagnosis (based on Nash et al. 2001 – also linked).

**Taxon profile pages** are the central vehicle in Symbiota for conveying information on a given taxon (Fig. 8). The editing interfaces allow information managers to: (1) provide synonyms and vernacular names; (2) view all annotated images (collection vouchers, specimens photographed *in situ*); (3) specify the sequence in which images appear on species profile pages; (4) add new images (either stored natively or just linked to the portal); and (5) add a (typically brief) natural language description of the taxon's morphological characters, including notes on natural history and distribution. Multiple feature-based descriptions can be added (original, revision, etc.), with links to source repositories for primary taxonomic literature such as the Biodiversity Heritage Library or the Flora of North America (1993).

Finally, **biotic inventories** are products generated by experts or expert teams and may integrate information entered, edited, and maintained in all other modules towards a regional flora, fauna, or both. Dynamically generated as well as static species checklists may be included. Static species lists give researchers complete and continuous control over species composition, taxonomic placements, region specific comments, and voucher assignments. One of the benefits of a virtual flora or fauna environment is the ability to establish all-inclusive species lists as well as a multitude of smaller lists covering more limited regions within the overall area of study.

### Current use and acceptance

As of May, 2014, 18 Symbiota portals are live and actively maintained on-line, representing more than 350 individual collections and a wide range of organismal groups (Table 1). SEINet, the oldest consortium and active since 2001, started out as a community portal for collections in the southwestern United States, focusing initially on lichens, vascular plants, pollen, and limited vertebrate and invertebrate data. SEINet was the first portal converted into a Symbiota portal in 2008. Over the past years, new communities have emerged and others have expanded their portal themes to include many North American collections some of which now have a world-wide scope. Other portals concentrate on select Neotropical and South American regions. Taxonomic coverage has also increased to include macroalgae, bryophytes, fungi, and some zoological groups (e.g., arthropods and vertebrates) where well established alternative platforms exist (Constable et al. 2010).

Many Symbiota portals are experiencing rapid growth in collection and occurrence record numbers, due in part to digitization projects supported by the NSF-ADBC program. Jointly these numbers and trends speak clearly of an increasing acceptance of Symbiota's bottom-up approach and flexibility in creating regionally or taxonomically themed biodiversity data communities.

### Future directions

Symbiota plays a pivotal role in North America in mobilizing small- to medium-sized natural history collections to enable voucher-based biodiversity research. Beyond recent funding successes, its impact is most significantly manifested in the creation, maintenance, and continuous expansion of strong portal communities over many years. This success has been due both to Symbiota's integrated and stable web-based CMS and the ability to effectively promote and support self-motivating collaborations at various regional and taxonomic scales. The balance struck in establishing fixed practices for data integration while remaining responsive to specific needs of research teams is also important in this context. Symbiota maintains its niche in the biodiversity informatics realm through extensive collaborations and integration of functionalities developed by other projects, thus allowing its core modules to interact functionally and share data with many other systems.

Sustaining and growing this role is a challenge with scientific, technical, and socio-economic dimensions that will remain relevant as long as the primary sources of support are competitive innovation and research grants. Like any developing biodiversity data platform, Symbiota is bound by the requirement to invest into continuous, adequate information technology and personnel infrastructure to remain useful (O'Brien 2010; NSF – National Science Foundation 2013; Stahlecker and Kroll 2013). With the user community growing, Symbiota must develop suitable business models that will allow portals to continue providing services by achieving sufficient revenue for sustaining the underlying infrastructure from the hub level down to the individual contributor (Penev et al. 2008; Beagrie et al. 2010; Triebel et al. 2012). For the underlying infrastructure this may include incorporating more 'off-the-shelf' software components the will simplyfy and distribute code maintenance. Many software solution have become available during the development of Symbiota which will have to be evaluated for their ease of integration, e.g., user management and security, certain modules from other content management systems.

Because interconnecting taxonomic information is an essential part of Symbiota's advanced functionality, another scalability challenge is the management of taxonomic thesauri over time and across regions. Alternative taxonomic perspectives may receive endorsement from separate portal communities whose areas of coverage overlap at least in part. Such situations are well illustrated by a succession of floristic treatments in the Southern and Mid-Atlantic United States (Franz et al. 2008; Weakley 2012; Franz et al. 2014a), or by persistent differences in conservation priorities due to an adherence to alternative species concepts in Mexican birds (Peterson and Navarro-Sigüenza 1999; Navarro-Sigüenza and Peterson 2004). At present Symbiota allows such conflicts to be expressed through different valid/synonymous *name* mappings between two classifications. However an implementation of *taxonomic concepts* and concept mappings can provide better data integration for the long term (Franz and Peet 2009; Franz and Cardona-Duque* 2013; Chen et al. 2014).

Readers interested in establishing new portals or joining existing portals can find more detailed, step-by-step instructions on the Symbiota software project website at http://symbiota.org.

## Web location (URIs)

Homepage: http://symbiota.org/

Download page: http://sourceforge.net/projects/symbiota/

## Technical specification

Platform: platform independent

Programming language: PHP, AJAX, JavaScript, jQuery

Operational system: Apache HTTP Web Server, or any web server with PHP

Interface language: English

## Repository

Type: SVN

Browse URI: http://sourceforge.net/projects/symbiota

## Usage rights

### Use license

Other

### IP rights notes

GNU General Public License, Version 2

## Figures and Tables

**Table 1.** List of Symbiota portal names and themes (in alphabetical order), URLs, and numbers of participating portal collections. Numbers generated in mid May, 2014.

**#**	**Portal Name and Theme**	**Portal URL**	**Collections**
1	CNABH – Consortium of North American Bryophyte Herbaria	http://bryophyteportal.org	55
2	CNALH - Consortium of North American Lichen Herbaria	http://lichenportal.org	58
3	CNH – Consortium of Northeastern Herbaria Portal	http://portal.neherbaria.org/	31
4	CoTRAM – Cooperative Taxonomic Resource for Amer. Myrtaceae	http://cotram.org/	6
5	Herbario Virtual Austral Americano	http://herbariovaa.org/	8
6	IRHN – Intermountain Region Herbarium Network*	http://intermountainbiota.org/	11
7	MABA – Madrean Archipelago Biodiversity Assessment – Fauna	http://www.madrean.org/symbfauna/	12
8	MABA – Madrean Archipelago Biodiversity Assessment – Flora*	http://www.madrean.org/symbflora/	22
9	Macroalgal Herbarium Consortium Portal	http://macroalgae.org	21
10	MyCoPortal - Mycology Collections Data Portal	http://mycoportal.org	39
11	NANSH – North American Network of Small Herbaria*	http://nansh.org/	11
12	Neotropical Arthropod Portal	http://symbiota.org/neotrop/entomology	5
13	Neotropical Flora Portal	http://symbiota.org/neotrop/plantae	11
14	Northern Great Plains Regional Herbarium Network*	http://ngpherbaria.org/	21
15	SCAN – Southwest Collections of Arthropods Network	http://symbiota1.acis.ufl.edu/scan	21
16	SEINet – Southwest Environmental Information Network	http://swbiodiversity.org/	25
17	SERNEC - Southeast Regional Network of Expertise and Collections*	http://sernecportal.org/portal/	7
18	Smithsonian Tropical Research Institute – Vertebrate Portal	http://symbiota.org/stri/verts/	12
